# Mechanical models and measurement methods of solid stress in tumors

**DOI:** 10.1007/s00253-024-13211-5

**Published:** 2024-06-06

**Authors:** Yingwei Bi, Jiacheng Jin, Rui Wang, Yuxin Liu, Liang Zhu, Jianbo Wang

**Affiliations:** 1 Department of Urology, First Affiliated Hospital, Dalian Medical University, Zhongshan Road 222, Dalian, 116011 China; 2 Dalian University of Technology, Linggong Road 2, Dalian, 116081 China; 3 Dalian Medical University, Lvshun South Road 9, Dalian, 116041 China

**Keywords:** Solid stress, Mechanical model, Tumor, Microenvironment

## Abstract

**Abstract:**

In addition to genetic mutations, biomechanical factors also affect the structures and functions of the tumors during tumor growth, including solid stress, interstitial fluid pressure, stiffness, and microarchitecture. Solid stress affects tumors by compressing cancer and stromal cells and deforming blood and lymphatic vessels which reduce supply of oxygen, nutrients and drug delivery, making resistant to treatment. Researchers simulate the stress by creating mechanical models both in vitro and in vivo. Cell models in vitro are divided into two dimensions (2D) and three dimensions (3D). 2D models are simple to operate but exert pressure on apical surface of the cells. 3D models, the multicellular tumor spheres, are more consistent with the actual pathological state in human body. However, the models are more difficult to establish compared with the 2D models. Besides, the procedure of the animal models in vivo is even more complex and tougher to operate. Then, researchers challenged to quantify the solid stress through some measurement methods. We compared the advantages and limitations of these models and methods, which may help to explore new therapeutic targets for normalizing the tumor’s physical microenvironment.

**Key points:**

•*This is the first review to conclude the mechanical models and measurement methods in tumors.*

•*The merit and demerit of these models and methods are compared.*

•*Insights into further models are discussed.*

## Introduction

The initiation and progression of tumors are generally considered to originate from mutations in genes, which result in abnormalities of cell proliferation, differentiation, and death. In fact, as tumors grow, they may interact with the surrounding microenvironment (including other tumor cells, normal cells, extracellular matrix, and interstitial fluid) due to the limited growing space, which exerts an effect on not only biological but also physical traits. The interaction may change the tumor structure and further affect the functions and regulate the malignant progression. There is increasing evidence indicating that tumor microenvironment plays a pivotal role during the process. Researchers divided these physical properties into four components, including solid stress, interstitial fluid pressure, material properties, and physical microarchitecture (Nia et al. 2020b). Biological effects have been studied for many years. However, the biomechanics of tumors is less explored because of the complicated internal microenvironment. Solid stress is exerted by the extracellular matrix and generated by cellular growth and remodeling (Helmlinger et al. 1997; Seano et al. 2019). It constantly accumulates in tumors with the rapid proliferation of cancer cells coping with the tumor microenvironment, which is in turn exerted on the surrounding normal tissue. Solid stress affects tumor pathophysiology mainly in two ways: directly by compressing cancer and stromal cells and indirectly by deforming blood and lymphatic vessels (Jain et al. 2014) and neuronal damage (Seano et al. 2019). Both are involved in the initiation and progression of tumors and compressing tumor blood vessels which reduces supply of oxygen, nutrients and drug delivery is resistant to the treatment (Chen et al. 2022; Nia et al. 2020a; Seano et al. 2019; Wang et al. 2022). Therefore, figuring out the properties of solid stress may provide us with new strategies for the treatment of cancers. Here we conclude several mechanical models and measurement methods in some tumors.

## Mechanical models

### 2D models in vitro

**Fig. 1:**
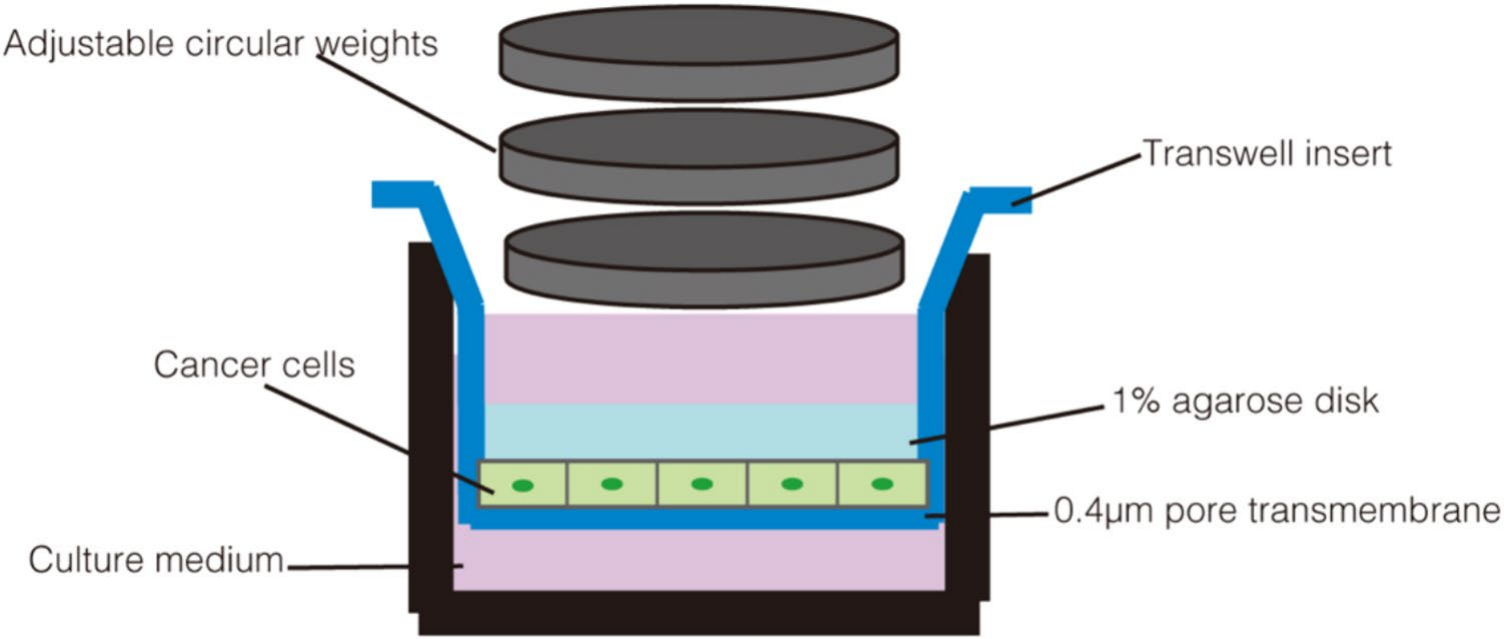
A pressure device of 2D model. Cancer cells are planted on the transmembrane of a 0.4 μm transwell insert. Adjustable circular weights are applied on a 1% agarose disk which is on the top of the cells. 1 ml and 1.5 ml media are added to the top and bottom chambers, respectively

We divide the in vitro mechanical models into two dimensions (2D) and three dimensions (3D) according to different ways of cell culture. The typical approach to exploring the effects of solid stress on the cytoskeleton in 2D models is to confine cells by physical contact between the apical surface of the cells and another solid surface (Fig. 1). The compressed surface can be a flat surface with less hardness, such as polydimethylsiloxane (PDMS) (He et al. 2018; Le Berre et al. 2014), agarose (Aureille et al. 2019; Taubenberger et al. 2019), or greater hardness, such as glass plates (Caille et al. 2002; Peeters et al. 2005). Our team used this model to explore the effects of growth-induced solid stress on cancer cell morphogenesis, epithelial-mesenchymal transition (EMT), and acquisition of a stemness phenotype by co-culturing renal cell carcinoma cells and T cells (Chen et al. 2017).

**Fig. 2:**
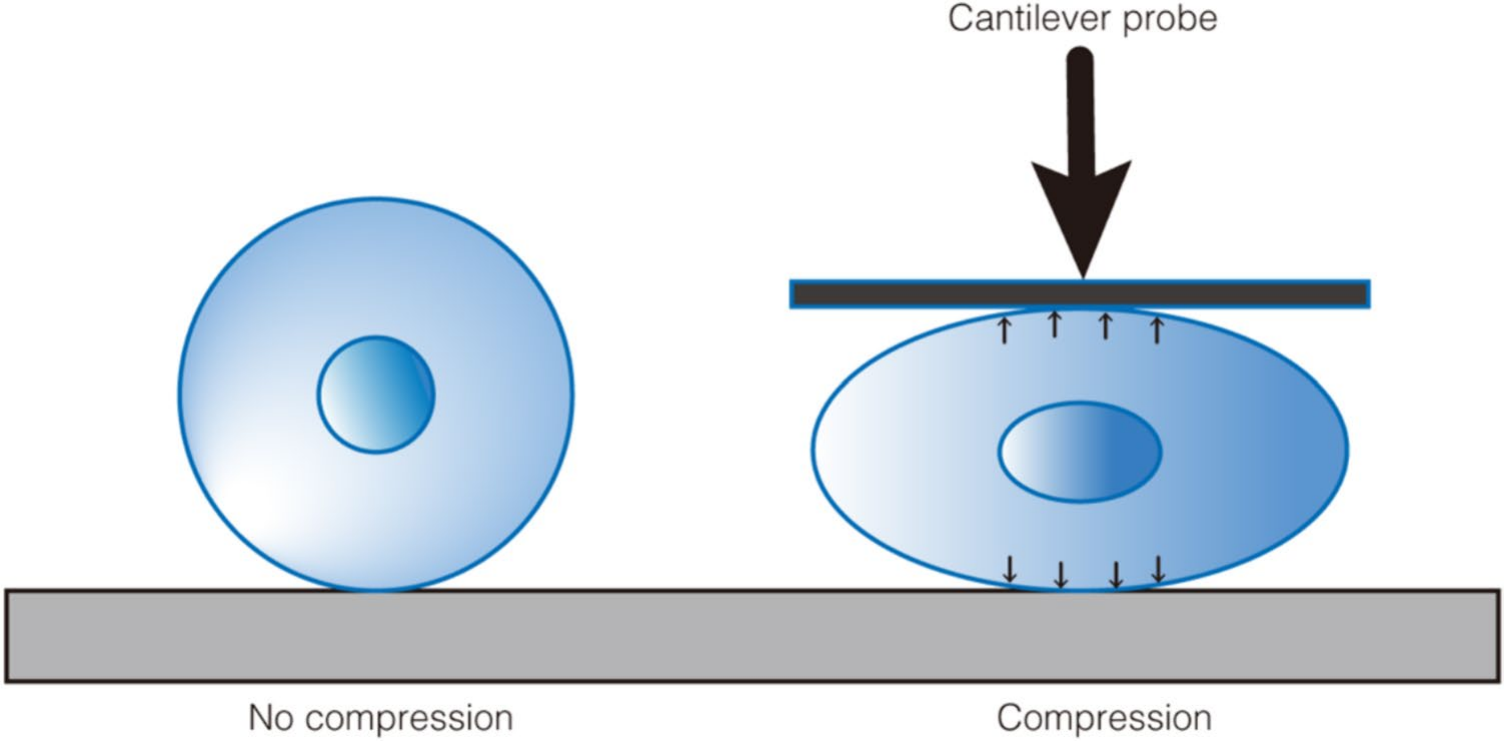
A cantilever probe model. A compressing cantilever probe driven by a piezoelectric actuator applies predefined different pressure axially to cells seeded onto glass slides

Li et al. (2018) created a method which was established to simultaneously quantify the elastic and viscoelastic properties of single cells based on atomic force microscopy (AFM) approach-reside-retract experiments. This research provides a novel way to quantify the mechanical properties of cells by AFM, which allows the investigation of the biomechanical behaviors of single cells from multiple aspects (Li et al. 2018). In addition to AFM, cantilever probes have also been used to apply stress to the apical surface of the cells (Ofek et al. 2009) (Fig. 2). Researchers used a video camera connected to a microscope to capture the changes of cytoskeleton compressive properties and recovery behavior of single cells.

The limitation of the 2D models is that the cells can only be stressed in the uniaxial direction at the same time, which is detached from the actual pathological state in the human body. However, the advantage is that cells can be probed in combination with high-resolution imaging, revealing important information about the cells’ response to mechanical stress.

### 3D models in vitro

**Fig. 3:**
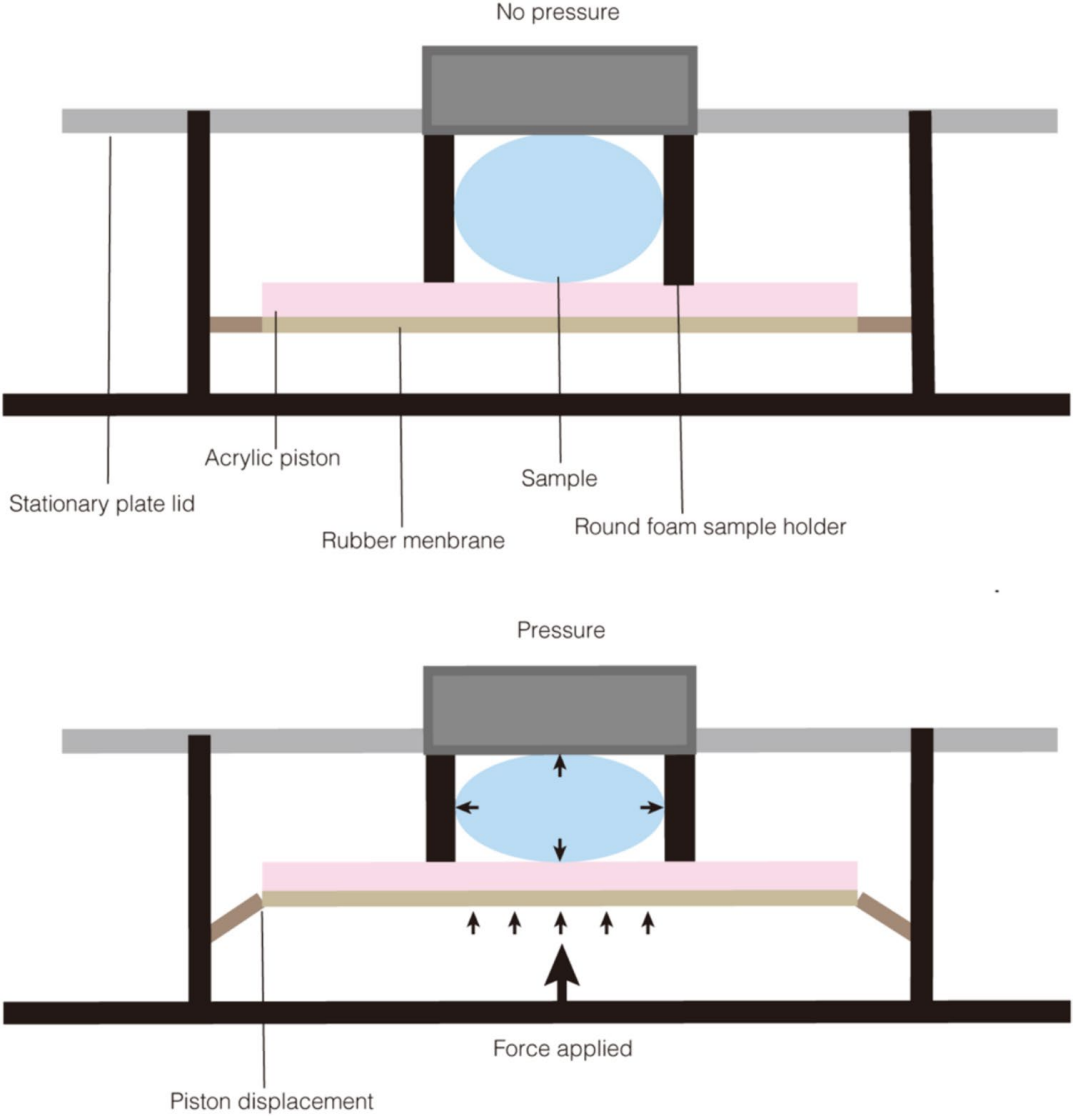
A pressure device of 3D model. The sample surrounded by round foam is put on the acrylic piston on rubber membrane (Upper). While applying pressure, the piston is displaced but the plate lid is not. The sample is subjected to pressure in all directions (Lower)

To explore a more physiological approach, researchers created 3D models of multicellular tumor spheres based on single-cell models (Fig. 3). In the tumor sphere models, uniaxial compression can inhibit cell growth and even induce programmed cell death (Cheng et al. 2009). However, when transitorily applied to a single cell, it may reverse the malignant phenotype, causing tumor cells to exhibit behaviors like normal cells (Ricca et al. 2018). It has been shown that gene expression profiles in 3D cultures reflect clinical profiles more accurately than those in 2D cultures (Hirschhaeuser et al. 2010). There are also various pressure media for the 3D tumor spheres, including rat tail collagen (Boyle et al. 2020), hydrogel (Marrella et al. 2019), agarose gel (Kalli et al. 2019; Lehtonen et al. 2023), and matrix gel (Dolega et al. 2021). However, the complete 3D control of uniform compression of tumor spheres has not been achieved in the previous multicellular tumor sphere models. Recently, Dolega et al. (2021) simulated solid stress according to the osmotic effect of dextran with different molecular weights to achieve uniform compression of single cells and tumor spheres. Dextran with small molecular weight can penetrate the extracellular matrix of the spheroids and exert osmotic pressure on individual cells. However, because dextran with large molecular weight cannot enter the extracellular matrix, it must act on the whole sphere. This allows comparison of different effects of solid stress on single cells and tumor spheres.

### Animal models in vivo

**Fig. 4:**
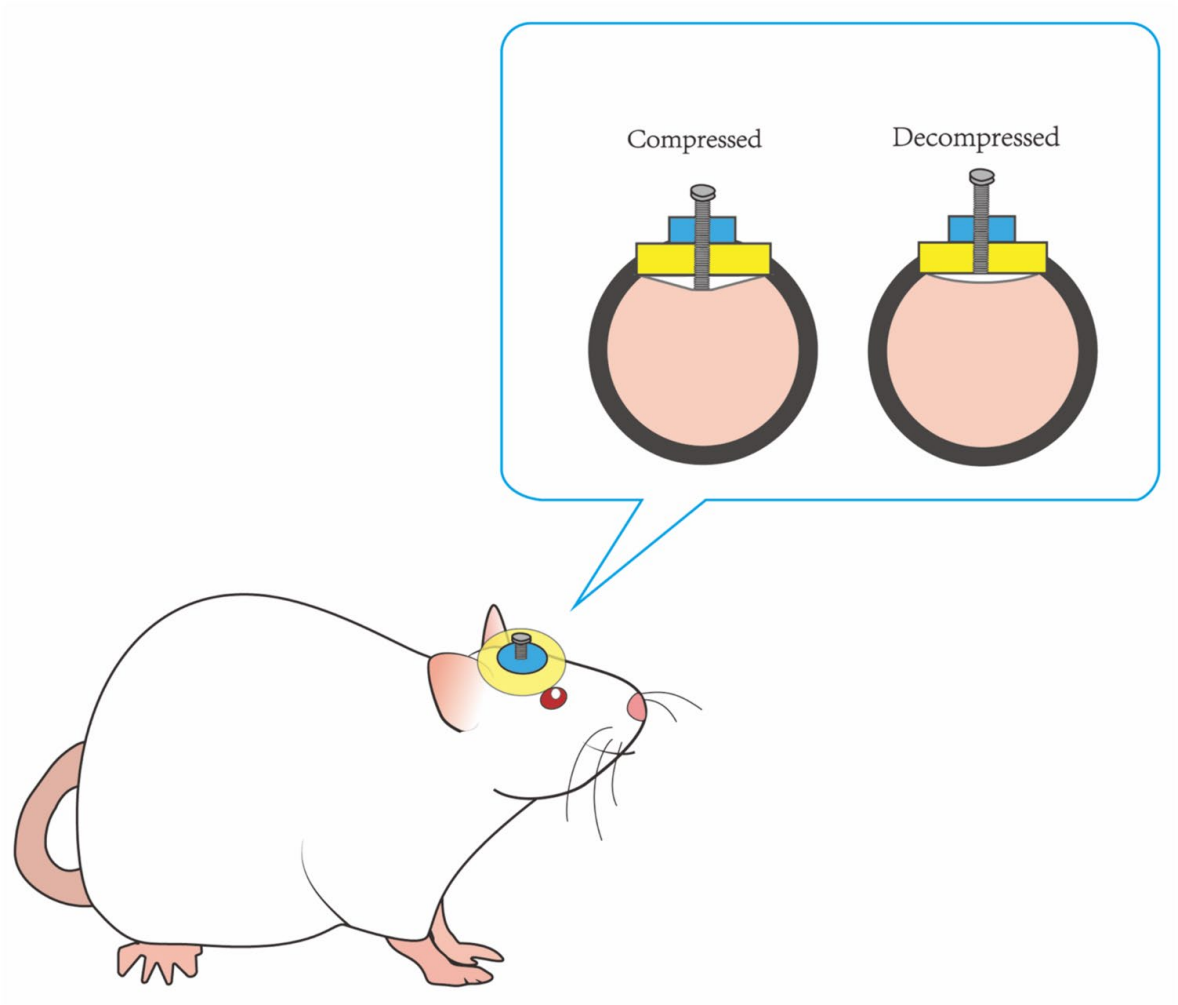
Rotatable screws device. A rotatable screw was implanted in the brain of the mouse

**Fig. 5:**
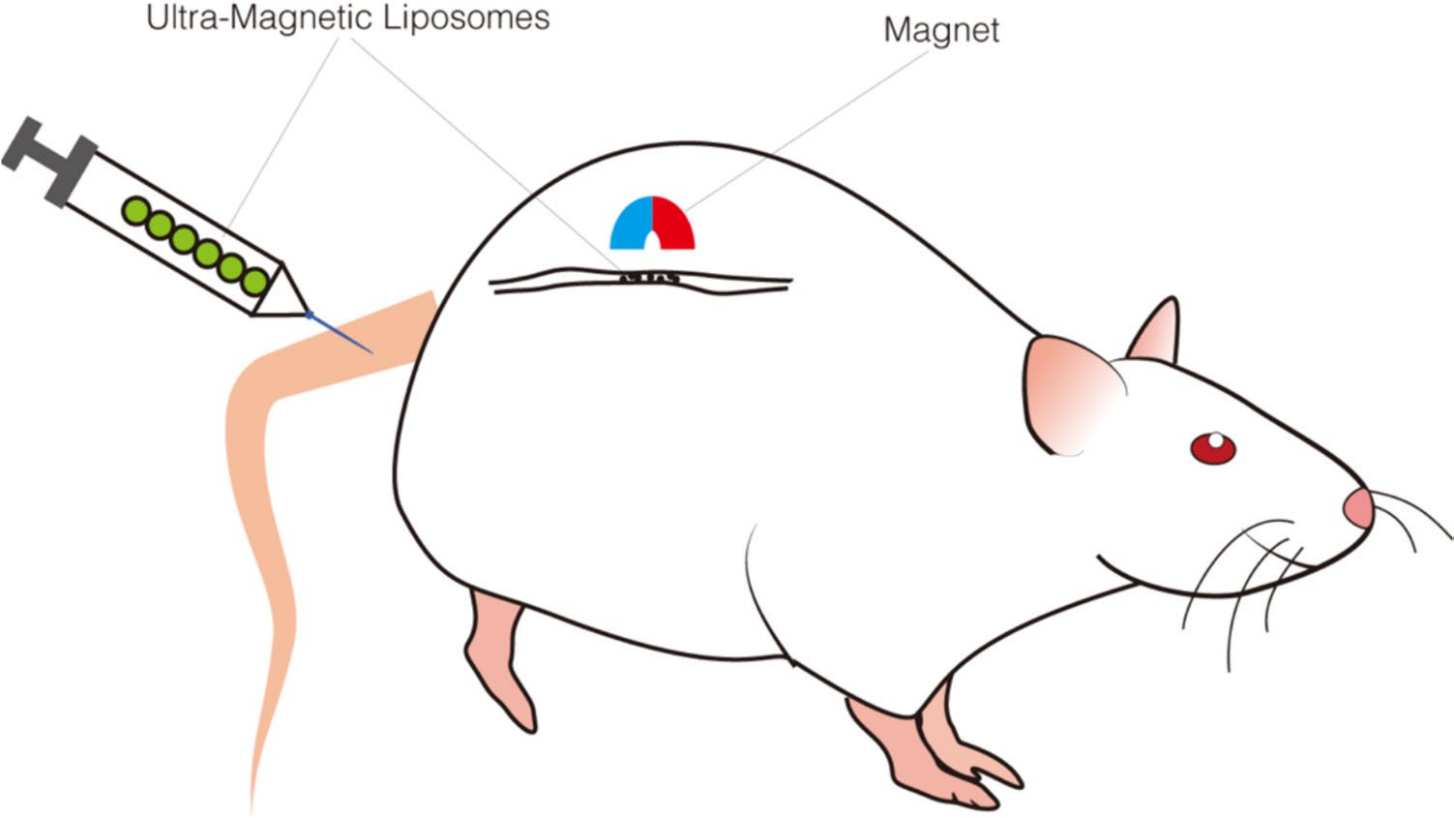
UML model. Subcutaneous insertion of the magnet on the back of the mice and in front of the distal colon, following UML injection in the lateral caudal vein of the mouse tail

Compared with the cell models in vitro, the procedure of the animal models in vivo is more complex and difficult to operate. Nia et al. developed an in vivo compression device that used rotatable screws to control compression and decompression of the cerebral cortex to simulate the solid mechanical force exerted by a growing tumor on surrounding brain tissues (Nia et al. 2020a) (Fig. 4). Fernandez-Sanchez et al. (2015) injected mice with ultra-magnetic liposomes and simultaneously inserted a magnet subcutaneously near the colon of mice to simulate the solid stress by using the magnetic force generated by the interaction between the magnet and the ultra-magnetic liposomes (UML) (Fig. 5). Then, they measured the tissue stress by magnet and quantitative Young’s modulus maps. The advantage of this method is that magnetic force can quantitatively simulate endogenous growth stress in early tumors without affecting the tissue hardness. Besides, it can also be applied to the mechanical study of drosophila embryos (Roper et al. 2018).

## Comparison of measurement methods of solid stress in tumors

### Measurement in normal tissues

In addition to tumors, solid stress is generated during normal growth and development. The shape of various cells changes significantly throughout the cell cycle due to mechanical action (Clark and Paluch 2011). In addition, the changes have been shown to play an important role in histomorphogenesis during tissue development. Studies showed that solid stress is widely distributed in arteries (Chuong and Fung 1986), heart (Omens et al. 1998), and brain (Xu et al. 2009). These tissues can be excised to retain growth-induced pressure. The removed tissues are then cut to release the stress, and the tissues deform in a measurable way. Xu et al. (2009) created a nonlinear finite element model for the mouse brain slice, using finite element software to estimate the magnitude of the residual stress.

### Measurement in tumors

Elevated solid stress, elevated interstitial fluid pressure, increased stiffness, and altered tissue microarchitecture are abnormal biomechanical characteristics of malignant tumors (Nia et al. 2020b). It is increasingly realized that these altered physical properties could affect the growth, differentiation, and invasion, even the treatment of solid tumors (Chaudhuri et al. 2020; Fernandez-Sanchez et al. 2015; Nia et al. 2016, 2020b). In contrast to the measurement of stiffness and fluid pressure of the tumor tissues, there are currently no high-resolution methods for quantifying solid stress in experimental animals or human tumors. Therefore, quantifying the solid stress in tumors which has gained attention in recent years is full of challenges (Kalli et al. 2019; Seano et al. 2019).

In 2003, Massachusetts General Hospital and Harvard Medical School jointly proposed a mathematical model of tumor growth in a finite elastic environment, which calculated the stress within the tumor and the surrounding media for the first time, and showed that the size of tumor cells decreases with increasing solid stress in the tumor spheres (Roose et al. 2003).

Another measuring method of solid stress (residual stress) in biological tissues is to use the deformation of fluorescent oil droplets (Campas et al. 2014). Andreas G. et al. developed a finite elements model to calculate growth-induced solid stress by simulating the opposite tissue deformation from the swollen tumor state to the non-swollen state and applied it to the breast, pancreatic, and fibrosarcoma tumor models (Hadjigeorgiou and Stylianopoulos 2023). The tumor microenvironment is continuously modified and remodeled due to tumor growth, and it is not clear which appropriate quantitative method should be chosen to measure deformations of solid tumors, because the material is characteristic and constantly changing (Ehlers et al. 2022; Grillo et al. 2019).

Although these methods can measure the stress generated by cells during embryonic morphogenesis and tissue development, it has some limitations. Firstly, the solid stress is indirectly revealed based on the deformation of incompressible oil droplets, which would not deform or change volume when exposed to solid stress equilibria in all directions. Secondly, the method is based on optical measurements of the droplet geometry; thus, the maximum measurement depth is controlled by the operating distance of the optical microscope, which is usually about 100–400 μm.

Uncovering the molecular mechanisms of mechanical force transduction has been hindered not only by limited exploration at the cellular level, but also at the sub-cellular level. Grashoff et al. (2010) used a calibrated biosensor which is called a single-molecule fluorescence force sensor to measure forces at the sub-cellular level across a specific protein, vinculin, in cells with pico-Newton (pN) sensitivity. Vinculin is a protein that links integrins to actin filaments and depends on force to function. However, whether the sensors apply to other molecules remains to be verified (Grashoff et al. 2010).

To avoid the limitations in the detection of cellular and subcellular stress by using fluorescent oil droplet injection and single-molecule fluorescence force sensors, researchers used material properties characterizing tissue stiffness to develop a mathematical model for calculating solid stress based on measured deformation (Du et al. 2019). However, this method is based on a partial cut of the spherical model of the tumor, which makes the precise release of solid stress and the measurement of deformation challenging. Besides, it is limited to the overall estimate of solid stress. In addition, this method is not suitable for the measurement of solid stress in small tumors and tumors in situ, and the resulting single-volume measurement does not highlight the heterogeneity of these stresses in different parts of the tumors. Based on releasing solid stress within tumors in a controlled manner and then calculating the stress-induced deformation by high-resolution ultrasound or light microscopy, Nia et al. (2018) and Nia et al. (2016) proposed three different experimental techniques and appropriate mathematical models to quantify solid stress and elastic potential energy in solid tumors. The three experimental techniques are (1) 2D mapping of solid stress in tumors (plane-cut method): This method allows us to distinguish the area with tensile stress (mainly in the periphery) from the central area of the tumor, which is mainly subjected to compressive stress, revealing the 2D spatial distribution of solid stress. (2) Sensitive measurement of small solid stress in small tumors (slice method): It provides a sensitive method to quantify low levels of solid stress in small samples, such as small metastases in mice. (3) Quantification of solid stress in situ tumors (needle biopsy): Solid stress can be estimated in situ, preserving the influence of the tumor microenvironment on the solid stress within the tumor. Besides, Nia et al. (2020a) recently developed a compression device in vivo that mimics solid stress placed on surrounding brain tissues by growing tumors and quantified solid stress both inside and outside tumors in the brain by using needle biopsy.

Additionally, to quantify growth-induced stress, tumors were cut, and the stress relaxation as the extent of tumor opening was measured. Then, the measured tumor openings were combined with the estimated material properties of tumor tissue and Triantafyllos’s mathematical model (Stylianopoulos et al. 2012).

To avoid invasive methods, Dolega et al. (2017) created non-destructive cell-like microsensors which were polyacrylamide microbeads of well-defined elasticity, size, and surface covering with enable internalization within the cellular environment to locally quantify mechanical stress distribution in multicellular spheroids. Besides, Fovargue et al. (2020) considered solid and fluid stress as a whole part and presented a proof-of-concept method to infer the total tumor stress (fluid and solid stress) by detecting the stiffness of peritumoral tissue with MR elastography and exploiting nonlinear biomechanical models.

### Measurement by imaging

Among the examinations of brain tumors, magnetic resonance elastography (MRE) is a method for quantifying the non-invasive shear modulus (such as stiffness and viscosity) of tissues. It provides guidance information for brain tumor resection (Palakurthi et al. 2018). Traditional elastography can estimate stiffness based on linear elasticity, but the prediction of solid stress in tumors is still controversial. However, the stiffness and solid stress can be distinguished through the combination with the compression-stiffening mechanism (that is, the nonlinear effect of stiffness changes in tissues under compression) (Perepelyuk et al. 2016) and MRE (Fovargue et al. 2020), which provides a non-invasive method for measuring solid stress in tumors—compression MRE (Page et al. 2021). On this basis, Page’s team further confirmed that solid stress is an independent determinant of compression hardening rate in addition to collagen contents, cell structures, and tumor types (Page et al. 2021). Besides, Lucci et al. (2022) present a neuroimaging‑informed mathematical multiphase model to quantify the solid and fluid stresses induced by tumor proliferation by evaluating the impact of the growing tumor mass on the surrounding healthy tissue.

Besides brain tumors, an in vivo non-invasive imaging technique for predicting solid stress within spherical tumors was built in breast cancers through a small animal model (Islam et al. 2019). Then, Islam et al. proposed a mathematical model to estimate volumetric strain inside spherical tumors under external compression(Islam and Righetti 2019) and developed an analytical model that can predict the modulus of elasticity and Poisson’s ratio of tumors (Islam et al. 2020). In addition, a biomechanical, biphasic model for the anisotropic growth of spinal tumors was presented which was used to predict the evolution of solid stress and interstitial fluid pressure in intramedullary spinal tumors (Katsamba et al. 2020). A 2D biomechanical model was developed to predict solid stress and interstitial fluid pressure together with elasticity imaging techniques in tumors based on the biphasic assumption of the solid matrix and fluid phase of the tissues (Dwairy et al. 2023).

Imaging and measuring mechanical stress, such as compressive, frictional, and tensile stress, has always been a challenging goal in materials science. Mechanical response materials detect mechanical stress by exhibiting visible color changes in their microstructure, i.e., conjugated length of chromophore changes as stress changes through the breaking of chemical bonds (Polacchi et al. 2019). Previous studies have shown that sufficient mechanical stress intensity is needed to induce molecular scale structural changes that lead to color changes (Davis et al. 2009; Gostl and Sijbesma 2016; Wang et al. 2015). Therefore, achieving a response to “weak” mechanical stress limits its application in the biomedical field. Nakamitsu et al. (2021) designed an ultra-sensitive compression stress sensor, a PDA/DL integrated device, using the concept of “response cascade,” which is similar to the cascade reaction in cell signaling. The colorimetric measurement of weak compressive stress (10^0^ − 10^3^ Pa) enriches the measurement method of physical characteristics of tumors and fills the gap in the measurement of weak solid stress in tumors.

### Viscoelasticity influences measurement accuracy

Viscoelasticity refers to the property of materials that exhibit both viscous and elastic behavior when undergoing deformed. Viscoelastic materials, such as biological tissues and extracellular matrix (ECM), both deform slowly under constant stress (viscous behavior) and recover their shape after the stress is removed (elastic behavior) (Fan et al. 2024). This combination of properties allows viscoelastic materials to absorb and dissipate energy, which is critical for the function and integrity of biological tissues under mechanical loads, such as solid stress (Chaudhuri et al. 2020). In cell models, the viscoelastic properties of the ECM and the cells themselves can influence cellular behavior, including migration, proliferation, and differentiation (Fan et al. 2024). Viscoelastic elements in tissue can absorb and dissipate energy over time, reducing the solid stress experienced by cells and tissues, which can protect cells from mechanical damage and regulate cell signaling pathways that are sensitive to mechanical cues. Advanced glycation end-products produced by type 2 diabetes mellitus create a stress-relaxing viscoelastic niche in the liver ECM, leading to activated mechanical signals promoting liver cancer (Fan et al. 2024). Therefore, enhancive viscoelasticity may be a risk factor for the risk profile of hepatocellular carcinoma (HCC) in NASH/T2DM. MRE is a good way to quantify the viscoelasticity of HCC though it is an invasive method (Page et al. 2021). Furthermore, the role of Yap in vivo as an inducer of cancer progression in response to viscoelastic changes was elucidated in vitro(Brusatin et al. 2018; Elosegui-Artola et al. 2023; Lee et al. 2019). Understanding and incorporating viscoelastic properties into experimental models can therefore enhance the prediction accuracy of solid stress in tumors.

## Conclusions and future perspectives

The tumor microenvironment is complicated, not only biologically but physically. There are numerous factors which can influence the initiation, progression, metastasis, and even death. It is precisely because of the complex mechanical microenvironment inside the tumor that it is difficult to simulate in vitro; thus, there are relatively few studies on mechanics compared with biology. Besides, solid stresses, interstitial fluid pressure, material properties, and physical microarchitecture within tumors are constantly changing during the development of tumors. These mechanical properties have an impact on the initial and expansion of tumors on one hand; competitive interactions which are called “mechanical cell competition” are produced with sharply increasing proliferation, which contributes to compacting and eliminating the neighboring cells on the other hand (Levayer 2020). Therefore, the tumor microenvironment including adjacent cells, extracellular matrix, and interstitial fluid affects the cells extensively. Precisely because these effects are extremely hard to simulate, some physical research of tumors is limited, including mechanical models and measurement methods of various mechanical stress.

Traditionally, 2D and 3D models were compared in terms of cell growth, gene expression, and drug resistance. The results showed that compared with 2D models, cells grown on 3D models could form aggregates and spheroids, which increased the contact between cells on one hand and the resistance to dacarbazine and cisplatin on the other (Fontoura et al. 2020). More physiologically appropriate models need to be explored both in vivo and in vitro. Similarly, some measurement methods and tools need to be created to identify the internal pressure of clinical tumor specimens and even directly measure the tumor pressure in patients. Although alleviating solid stress and decompressing vessels might promote tumor growth by delivering more nutrients to cancer cells, some pieces of research have indicated that therapies can reduce solid stress and improve perfusion and delivery of drugs so as to achieve cancer treatment (Chen et al. 2020; Provenzano et al. 2012; Zhao et al. 2019). We are the first to conclude the mechanical models and measurement methods in tumors which may provide researchers new thoughts to create more novel models. In this way, we can more accurately intervene in abnormal tumor pressure, or a series of metabolic and related pathway changes caused by pressure, explore the relationship between physical and biological to restore the abnormality of the tumor’s physical microenvironment, inhibit the malignment behavior of the tumor, and provide novel targets for clinical tumor treatments.
